# Comparative assessment of the quality and reliability of cerebral infarction–related short-video health information on TikTok and Bilibili: A cross-sectional study

**DOI:** 10.1097/MD.0000000000049206

**Published:** 2026-06-12

**Authors:** Yifeng Xie, Yuzhang Liang, Haiyan Song, Yike Liu, Ya Na, Jiamin Gao, Ao Ying, Chunyan Chen

**Affiliations:** a Affiliated Bayannur Clinical College, Inner Mongolia Medical University, Bayannur, China; b Department of Neurology, Affiliated Bayannur Clinical College, Inner Mongolia Medical University, Bayannur, China.

**Keywords:** Bilibili, cerebral infarction, health communication, information quality, social media, TikTok

## Abstract

Short-video platforms are important sources of cerebral infarction–related health information, but the completeness, reliability, and platform differences of such content remain unclear. This study compared engagement, topic coverage, and information quality of cerebral infarction-related videos on TikTok and Bilibili. Using the keyword “cerebral infarction,” we screened the top 150 search results from each platform. Of 300 retrieved videos, 289 were included (TikTok: n = 146; Bilibili: n = 143). Video duration and engagement metrics were extracted, and uploaders were classified as doctors of health professions (DHPs), non-doctors of health professions (NDHPs), or individual users. Topic coverage was coded as not covered, partially covered, or completely covered. Information quality and reliability were assessed using GQS, mDISCERN, JAMA benchmarks, and VIQI. Mann–Whitney U, Kruskal–Wallis with Dunn–Bonferroni post hoc tests, and Spearman correlation analyses were performed. Content coverage was uneven. Epidemiology was absent in 85.8% of videos and completely covered in only 0.3%, whereas diagnosis was absent in 69.6% and completely covered in 10.0%. Etiology and clinical manifestations were more frequently addressed, with complete coverage in 61.2% and 37.7%, respectively. Bilibili videos were longer than TikTok videos, whereas TikTok videos showed higher likes, collections, comments, and shares (all *P* < .001). Platform differences were observed across all 4 quality instruments. TikTok videos had higher JAMA scores, whereas Bilibili videos had higher mDISCERN and VIQI scores; GQS also differed significantly despite a median score of 3.0 on both platforms. DHP-uploaded videos had higher quality scores than NDHP- or individual-user videos across all 4 instruments (all *P* < .001). Platform-stratified correlations between engagement and quality scores were weak, ranging from *r* = −0.257 to *r* = 0.139 on TikTok and from *R* = 0.066 to *R* = 0.298 on Bilibili. Cerebral infarction-related videos on TikTok and Bilibili showed modest overall quality and gaps in key actionable domains, particularly epidemiology and diagnosis. Engagement was a poor proxy for information quality. Stronger credibility labeling, source disclosure, and quality-aware recommendation strategies may improve short-video health information.

## 1. Introduction

Cerebral infarction is one of the leading causes of death and disability worldwide.^[1]^ Effective prevention and treatment depend heavily on the public’s correct understanding of the risk factors, typical symptoms, and the concept of the “time window” for treatment.^[2]^ Timely recognition of symptoms and rapid initiation of emergency procedures are crucial for reducing mortality and disability. However, public knowledge of cerebral infarction remains insufficient, underscoring the urgent need for accessible, understandable, and reliable health education to support early recognition and appropriate medical decisions.

In recent years, short video platforms such as Douyin (TikTok’s Chinese mainland counterpart) and Bilibili have rapidly developed into important channels for disseminating health information to the public in China.^[3]^ Short videos are characterized by fast dissemination, broad reach, intuitive presentation, and ease of sharing, all of which contribute to promoting health knowledge to a certain extent.^[4]^ However, the low threshold for content production, rapid information updates, and algorithm-driven distribution lead to significant variation in the quality and reliability of health information.^[5]^ The level of engagement often reflects the dissemination and entertainment attributes of the content, but does not necessarily indicate the accuracy, completeness, or transparency of medical information.^[6]^ In the context of cerebral infarction, where “time is brain,” if videos lack key recognition and diagnostic guidance or contain misleading information, it may delay medical care and pose potential risks.

Existing research on health information in short videos mainly focuses on a single platform or evaluation dimension. There is limited cross-platform comparative evidence on cerebral infarction-related videos in Chinese platforms, particularly a lack of comprehensive analysis that combines uploader types, content coverage structure, and multidimensional quality assessment.^[4]^ Therefore, from February 27 to March 2, 2026, this study systematically retrieved and screened cerebral infarction-related videos from TikTok and Bilibili, extracted engagement metrics and content coverage information, and evaluated the quality and reliability of the videos using GQS, modified DISCERN, JAMA, and VIQI. This study further compares the differences across platforms and uploader types and analyzes the correlations between engagement indicators and quality scores, providing empirical evidence for the need to optimize cerebral infarction health communication and platform governance.

## 2. Methods

### 2.1. Study design

This cross-sectional descriptive study evaluated the engagement characteristics, content coverage, and information quality of cerebral infarction-related short videos on 2 major Chinese short-video platforms, TikTok and Bilibili. Video retrieval, screening, data extraction, and rating were conducted between February 27 and March 2, 2026, using a predefined study protocol and standardized evaluation criteria to improve consistency and reduce subjective bias. This study followed the STROBE principles for cross-sectional research reporting.^[7]^

### 2.2. Data sources and retrieval strategy

We searched TikTok and Bilibili, 2 widely used Chinese short-video platforms, during the study period. In this manuscript, the platform referred to as TikTok corresponds to Douyin, the Chinese mainland counterpart of TikTok. To minimize the influence of personalized recommendation algorithms, all searches were performed using newly registered accounts in a relatively consistent search environment.^[4]^ The search was conducted using the keyword “cerebral infarction” only. Related terms such as “stroke” were not included, in order to maintain a uniform and reproducible search strategy across platforms. Videos were sorted according to each platform’s default search ranking, and the first 150 videos retrieved from each platform were selected for initial screening.^[8]^ For each platform, the search date, platform source, and retrieval conditions were recorded. This approach was designed to reflect the type of content most likely to be encountered by ordinary users under routine search conditions.^[9]^

### 2.3. Eligibility criteria and video screening

Videos were eligible for inclusion if they met all of the following criteria: they were related to cerebral infarction and contained health information concerning disease concepts, etiology or risk factors, clinical manifestations, diagnosis, treatment, prognosis, rehabilitation, or secondary prevention; they were playable and provided sufficient information for evaluation; and they were presented in Chinese or primarily in Chinese.

Videos were excluded if they were irrelevant to cerebral infarction despite relevant titles or labels; were duplicates, in which case only one version was retained; consisted primarily of entertainment or personal narrative content without substantive health information; were advertisements or marketing-oriented videos without meaningful medical education content; or lacked accessible key indicators, such as video duration or engagement metrics, or could not be played normally.

Screening was performed independently by 2 researchers with medical training, and disagreements were resolved through discussion with a third senior researcher from the Department of Neurology. Initial screening was based on titles, descriptions, and visible content, followed by full-video review for potentially eligible records.

### 2.4. Data extraction

For each included video, 3 researchers independently extracted data using a standardized data collection form, and discrepancies were resolved by cross-checking and discussion. Extracted variables included platform source, video duration, likes, collections/favorites, comments, and shares.

Uploader type was classified into 3 categories according to account identity and content characteristics: doctors of health professions (DHPs), non-doctors of health professions (NDHPs), and individual users. DHPs included physicians and other health professionals directly involved in medical care, such as neurologists and emergency physicians. NDHPs included accounts with health-related backgrounds that were not directly identified as physicians in relevant clinical specialties. Individual users referred to nonprofessional personal accounts without a clearly identifiable health professional background.

### 2.5. Content coverage assessment

Each video was assessed across 6 predefined content domains: epidemiology, etiology, clinical manifestations, diagnosis, treatment, and prognosis. Epidemiology referred to information on disease burden, frequency, high-risk groups, or population distribution. Etiology referred to causes or risk factors. Clinical manifestations referred to symptoms or warning signs. Diagnosis referred to symptom recognition, clinical evaluation, diagnostic tests, imaging, or diagnostic confirmation. Treatment referred to acute management, medication, intervention, rehabilitation, or related therapeutic strategies. Prognosis referred to outcomes, recurrence, disability, recovery, or long-term expectations.

For each domain, coverage was coded as not covered, partially covered, or completely covered, according to predefined criteria. “Partially covered” indicated that the topic was mentioned but incompletely explained, whereas “completely covered” indicated relatively comprehensive and clearly presented information within that domain.

### 2.6. Quality and reliability assessment

This study used multiple parallel tools to assess the quality and reliability of health information from different dimensions.

1. GQS (Global Quality Score) was used to assess the overall educational quality, flow, and usefulness of the video from the viewer’s perspective (Supplemental Digital Content 1).^[10]^
2. mDISCERN (Modified DISCERN) was used to evaluate the reliability of health information and whether the content supported balanced and trustworthy health decision-making (Supplemental Digital Content 2).^[11]^
3. JAMA (Journal of the American Medical Association) was used to assess basic source transparency and credibility, including authorship, attribution, disclosure, and currency (Supplemental Digital Content 3).^[12]^
4. Video Information and Quality Index (VIQI) was used to evaluate information delivery and presentation quality, including content flow, accuracy-related presentation, and production-related features (Supplemental Digital Content 4).^[13]^

Two researchers with medical training completed unified training before formal scoring and conducted pilot assessments to standardize interpretation of the rating tools. During the formal evaluation stage, videos were scored independently, and disagreements were resolved through discussion with a third senior researcher from the Department of Neurology. This process was used to improve rating consistency and reduce subjective variation.

### 2.7. Statistical analysis

Descriptive statistics were used to summarize video characteristics, content coverage, and quality assessment results. Continuous variables are presented as medians and interquartile ranges (IQRs), and categorical variables are presented as frequencies and percentages.

For comparisons between TikTok and Bilibili, continuous variables were analyzed using the Mann–Whitney *U* test. For comparisons among uploader groups (DHPs, NDHPs, and individual users), continuous variables were analyzed using the Kruskal–Wallis test. When the overall Kruskal–Wallis test was statistically significant, Dunn’s post hoc test with Bonferroni correction was performed for pairwise comparisons. Spearman rank correlation analysis was performed to assess associations between engagement indicators, video duration, and quality scores, including GQS, mDISCERN, JAMA, and VIQI. Spearman correlation coefficients and corresponding P values were reported.

All statistical analyses were performed using IBM SPSS Statistics version 27.0, and figures were generated using GraphPad Prism version 10.4. A 2-sided *P* < .05 was considered statistically significant.

### 2.8. Ethical considerations

This study analyzed only publicly accessible online videos and did not involve human participants, patient data, or identifiable personal information; therefore, ethics committee approval and informed consent were not required.

## 3. Results

### 3.1. Video characteristics

**Figure 1.**
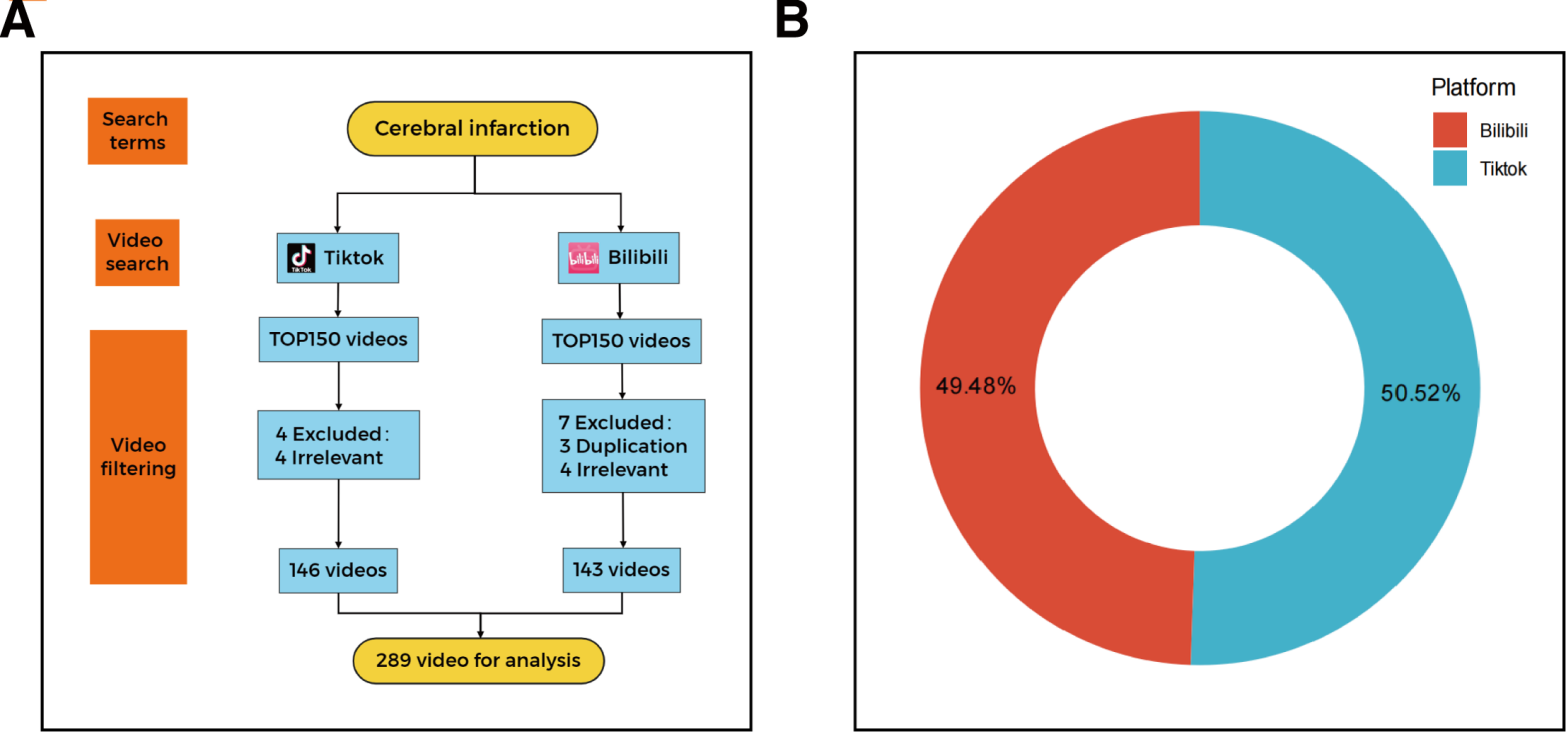
Study flow diagram and platform composition of included videos. (A) Flowchart of video identification, screening, exclusion, and final inclusion for analysis. “Irrelevant” refers to videos whose titles or labels suggested relevance to cerebral infarction but whose actual content did not provide cerebral infarction-related health information. (B) Proportion of included cerebral infarction-related videos from each platform (TikTok and Bilibili).

A total of 300 videos were initially retrieved, comprising the top 150 search results from TikTok and the top 150 from Bilibili. After screening, 289 videos met the eligibility criteria and were included in the final analysis, including 146 TikTok videos and 143 Bilibili videos. Eleven videos were excluded because they were duplicates or irrelevant to cerebral infarction. The screening process is shown in Figure 1. Among the prespecified exclusion criteria, only duplicate and irrelevant videos were encountered during the screening process.

Across all included videos, the median (IQR) number of likes was 368.0 (70.0–4557.0), collections was 316.0 (48.0–2283.0), comments was 29.0 (4.0–231.0), and shares was 140.0 (19.0–1133.0). The median video duration was 141 seconds (72–296). In terms of information quality, the median (IQR) scores were 3.0 (2.0–3.0) for GQS, 2.0 (2.0–3.0) for mDISCERN, 2.0 (1.0–2.0) for JAMA, and 7.0 (6.0–9.0) for VIQI. The overall characteristics and quality scores of the included videos are summarized in Table 1.

**Table 1:** Overall characteristics and quality scores of included videos.

Variables	Total (n = 289)
General information	
Video length, s, Median (Q1, Q3)	141.00 (72.00, 296.00)
Likes, Median (Q1, Q3)	368.00 (70.00, 4557.00)
Collections, Median (Q1, Q3)	316.00 (48.00, 2283.00)
Comments, Median (Q1, Q3)	29.00 (4.00, 231.00)
Shares, Median (Q1, Q3)	140.00 (19.00, 1133.00)
Video quality	
GQS score, Median (Q1, Q3)	3.00 (2.00, 3.00)
mDISCERN score, Median (Q1, Q3)	2.00 (2.00, 3.00)
JAMA score, Median (Q1, Q3)	2.00 (1.00, 2.00)
VIQI score, Median (Q1, Q3)	7.00 (6.00, 9.00)

GQS = Global Quality Score, JAMA = Journal of the American Medical Association, mDISCERN = modified DISCERN, Q1 = first quartile, Q3 = third quartile, VIQI = Video Information and Quality Index.

### 3.2. Content coverage

**Figure 2.**
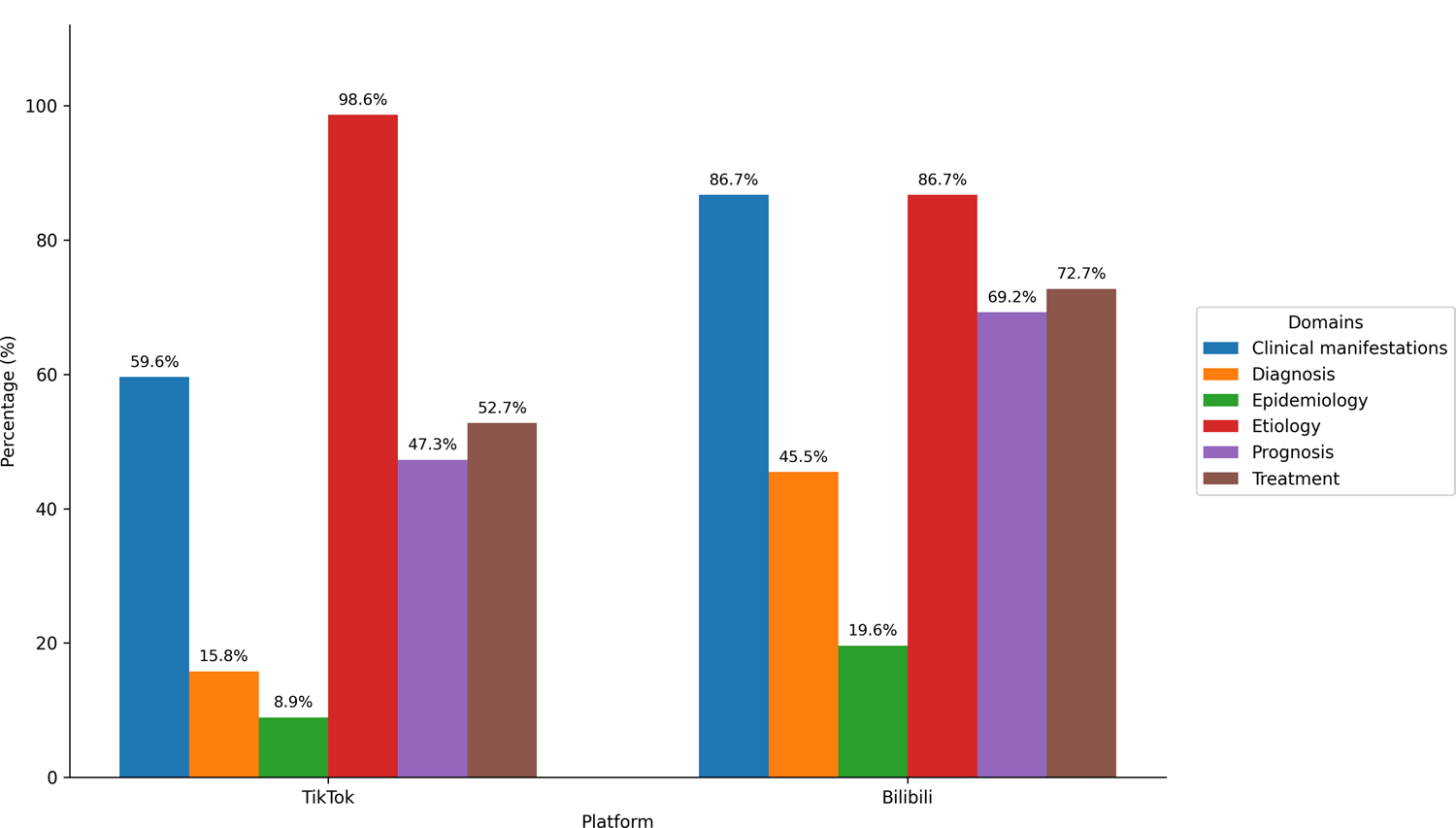
Comparison of topic coverage between TikTok and Bilibili. Bar chart showing the percentage of videos covering each predefined content domain, including epidemiology, etiology, clinical manifestations, diagnosis, treatment, and prognosis, on TikTok and Bilibili.

Content coverage was uneven across the included videos (Table 2 and Fig. 2). Etiology was the most frequently addressed domain: only 7.3% of videos did not cover this topic, whereas 31.5% provided partial coverage and 61.2% provided complete coverage. Clinical manifestations were also relatively well represented, with 7.2% of videos not covering this domain, 35.3% providing partial coverage, and 37.7% providing complete coverage.

**Table 2:** Coverage of key cerebral infarction content domains in videos.

Content domain	Not covered	Partially covered	Completely covered
Epidemiology, n (%)	248 (85.8)	40 (13.9)	1 (0.3)
Etiology, n (%)	21 (7.3)	91 (31.5)	177 (61.2)
Clinical manifestations, n (%)	21 (7.2)	102 (35.3)	109 (37.7)
Diagnosis, n (%)	201 (69.6)	59 (20.4)	29 (10.0)
Treatment, n (%)	108 (37.4)	79 (27.3)	102 (35.3)
Prognosis, n (%)	121 (41.9)	96 (33.2)	72 (24.9)

n (%) = number (percentage).

By contrast, epidemiology and diagnosis were the least represented domains. Epidemiology was not covered in 85.8% of videos, was partially covered in 13.9%, and was completely covered in only 0.3%. Diagnosis was absent in 69.6% of videos, partially covered in 20.4%, and completely covered in 10.0%. Treatment was not covered in 37.4% of videos, partially covered in 27.3%, and completely covered in 35.3%. Prognosis was absent in 41.9% of videos, partially covered in 33.2%, and completely covered in 24.9%.

### 3.3. Platform comparison

Table 3 presents the comparison of engagement, content coverage, and quality scores between TikTok and Bilibili. In terms of engagement, TikTok videos showed significantly higher interaction than Bilibili videos. The median (IQR) number of likes was 3200.5 (395.0–12,904.8) on TikTok versus 107.0 (17.0–351.5) on Bilibili; collections were 1150.0 (109.5–5854.3) versus 132.0 (25.5–588.5); comments were 130.5 (26.8–634.5) versus 6.0 (1.0–30.5); and shares were 809.5 (65.5–6100.5) versus 46.0 (10.0–205.5), respectively.

**Table 3:** Comparison of engagement metrics and quality scores between platforms.

Variables	Bilibili (n = 143)	TikTok (n = 146)	*P*
General information			
Video length, s, Median (Q1, Q3)	205.00 (115.50, 478.50)	110.00 (50.25, 167.75)	<.001
Likes, Median (Q1, Q3)	107.00 (17.00, 351.50)	3200.50 (395.00, 12,904.75)	<.001
Collections, Median (Q1, Q3)	132.00 (25.50, 588.50)	1150.00 (109.50, 5854.25)	<.001
Comments, Median (Q1, Q3)	6.00 (1.00, 30.50)	130.50 (26.75, 634.50)	<.001
Shares, Median (Q1, Q3)	46.00 (10.00, 205.50)	809.50 (65.50, 6100.50)	<.001
Video quality			
GQS score, Median (Q1, Q3)	3.00 (2.00, 4.00)	3.00 (2.00, 3.00)	<.001
mDISCERN score, Median (Q1, Q3)	2.00 (2.00, 3.00)	2.00 (0.50, 2.00)	.039
JAMA score, Median (Q1, Q3)	1.00 (1.00, 2.00)	2.00 (2.00, 2.00)	<.001
VIQI score, Median (Q1, Q3)	8.00 (6.00, 11.00)	7.00 (4.50, 7.00)	<.001

GQS = Global Quality Score, JAMA = Journal of the American Medical Association, mDISCERN = modified DISCERN, Q1 = first quartile, Q3 = third quartile, VIQI = Video Information and Quality Index.

**Figure 3.**
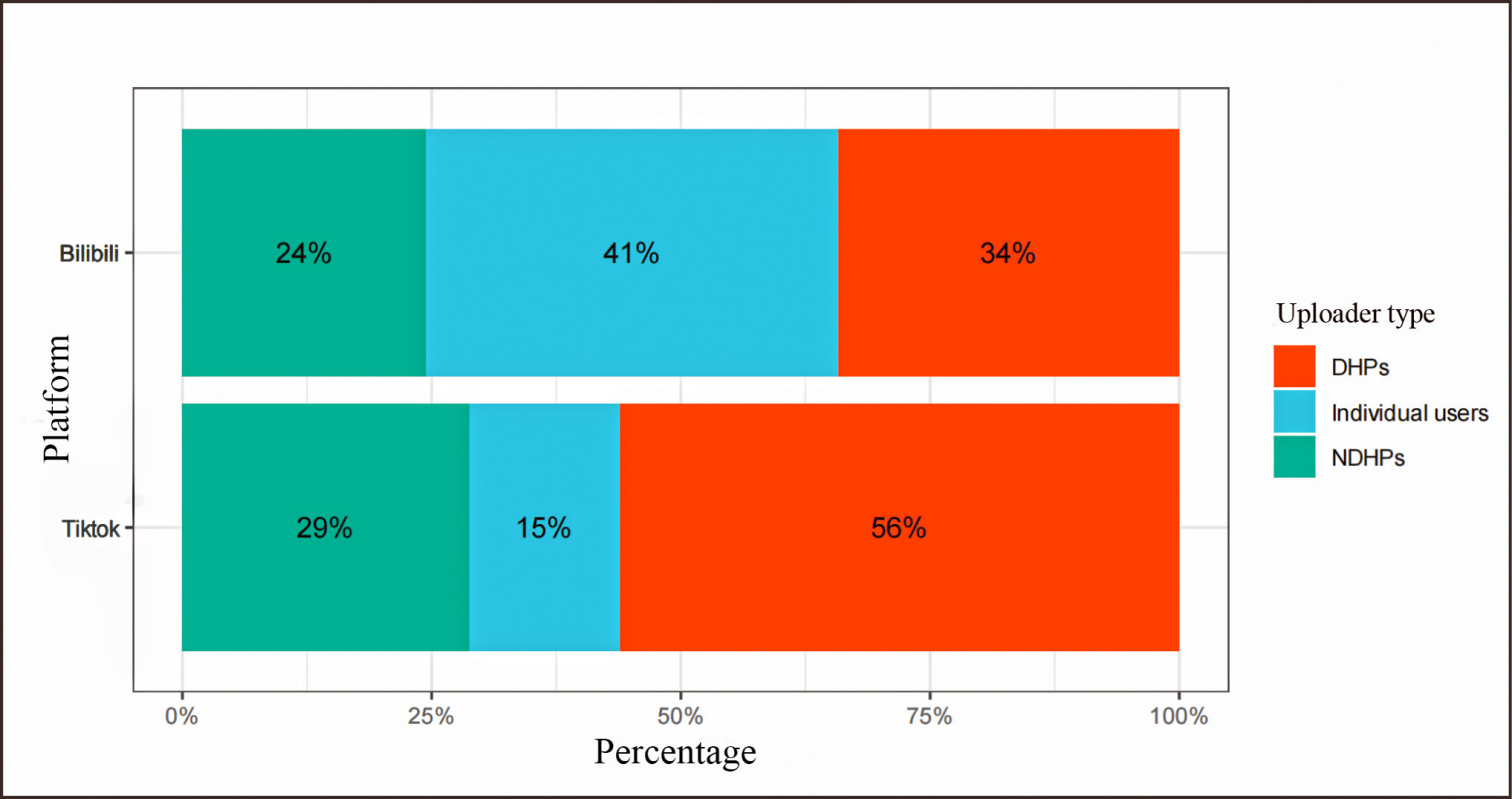
Platform differences in video quality and reliability scores. (A–D) Kernel density distributions of Global Quality Score (GQS), modified DISCERN (mDISCERN), Journal of the American Medical Association (JAMA), and Video Information and Quality Index (VIQI) scores for TikTok and Bilibili. (E–H) Violin plots comparing the score distributions of GQS, mDISCERN, JAMA, and VIQI between platforms; embedded boxplots indicate the median and interquartile range, and brackets indicate between-platform statistical comparisons.

With regard to information quality, significant between-platform differences were observed across all 4 instruments (GQS, *P* < .001; mDISCERN, *P* = .039; JAMA, *P* < .001; VIQI, *P* < .001) (Table 3 and Fig. 3). For GQS, both platforms had a median score of 3.0, although the score distributions differed significantly. TikTok videos had higher JAMA scores than Bilibili videos, whereas Bilibili videos had higher median mDISCERN and VIQI scores. These findings indicate that the 2 platforms showed different quality profiles rather than a uniform advantage of one platform across all assessed dimensions. The corresponding score distributions are shown in Figure 3.

**Figure 4.**
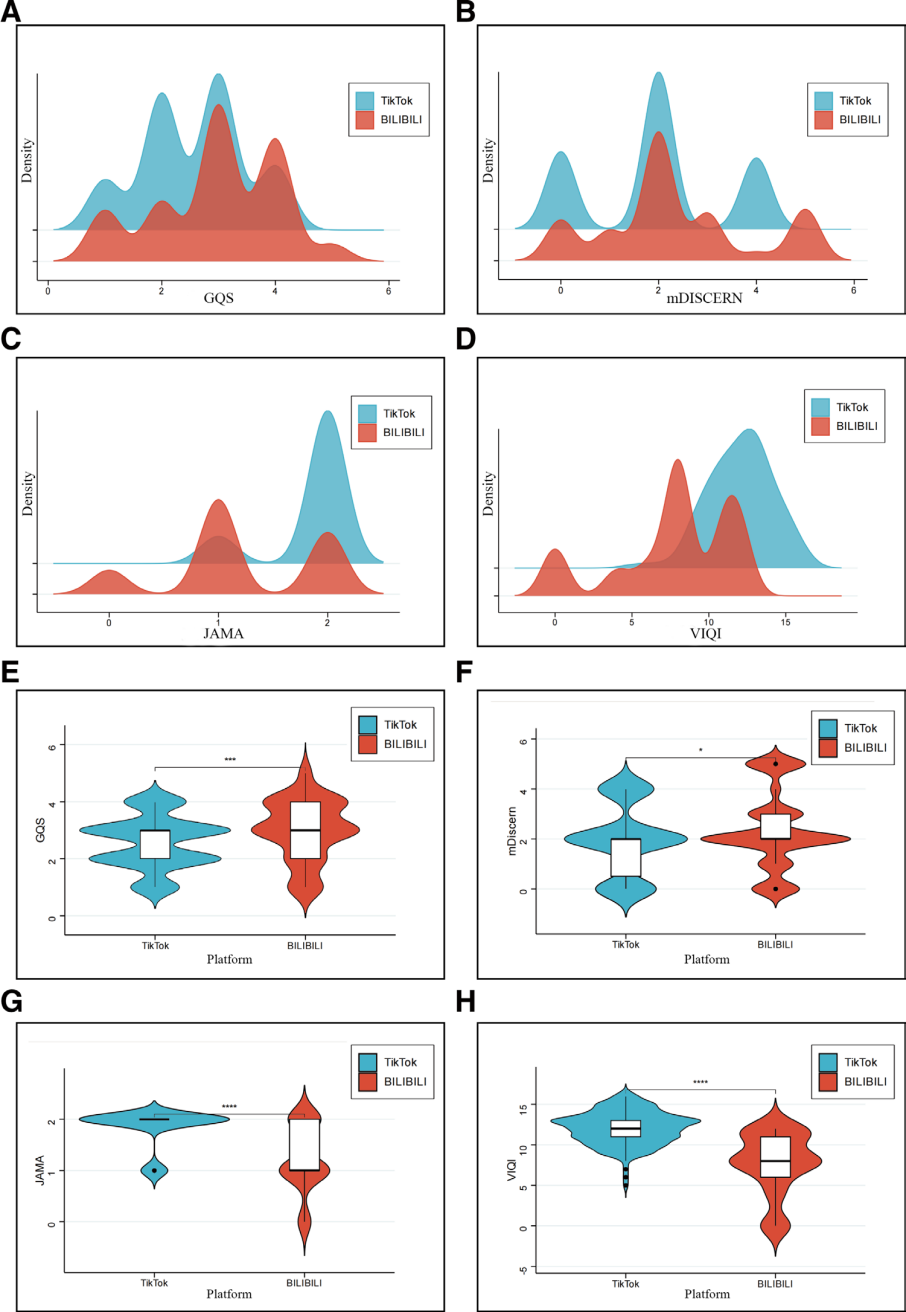
Distribution of uploader types across platforms. Stacked bar chart showing the percentage distribution of uploader types on TikTok and Bilibili, including doctors of health professions (DHPs), non-doctors of health professions (NDHPs), and individual users.

Uploader composition also differed between platforms (Fig. 4). On Bilibili, individual users accounted for the largest proportion of uploads (41%), followed by DHPs (34%) and NDHPs (24%). On TikTok, DHPs represented the largest uploader group (56%), followed by NDHPs (29%) and individual users (15%).

### 3.4. Comparisons by uploader type

**Figure 5.**
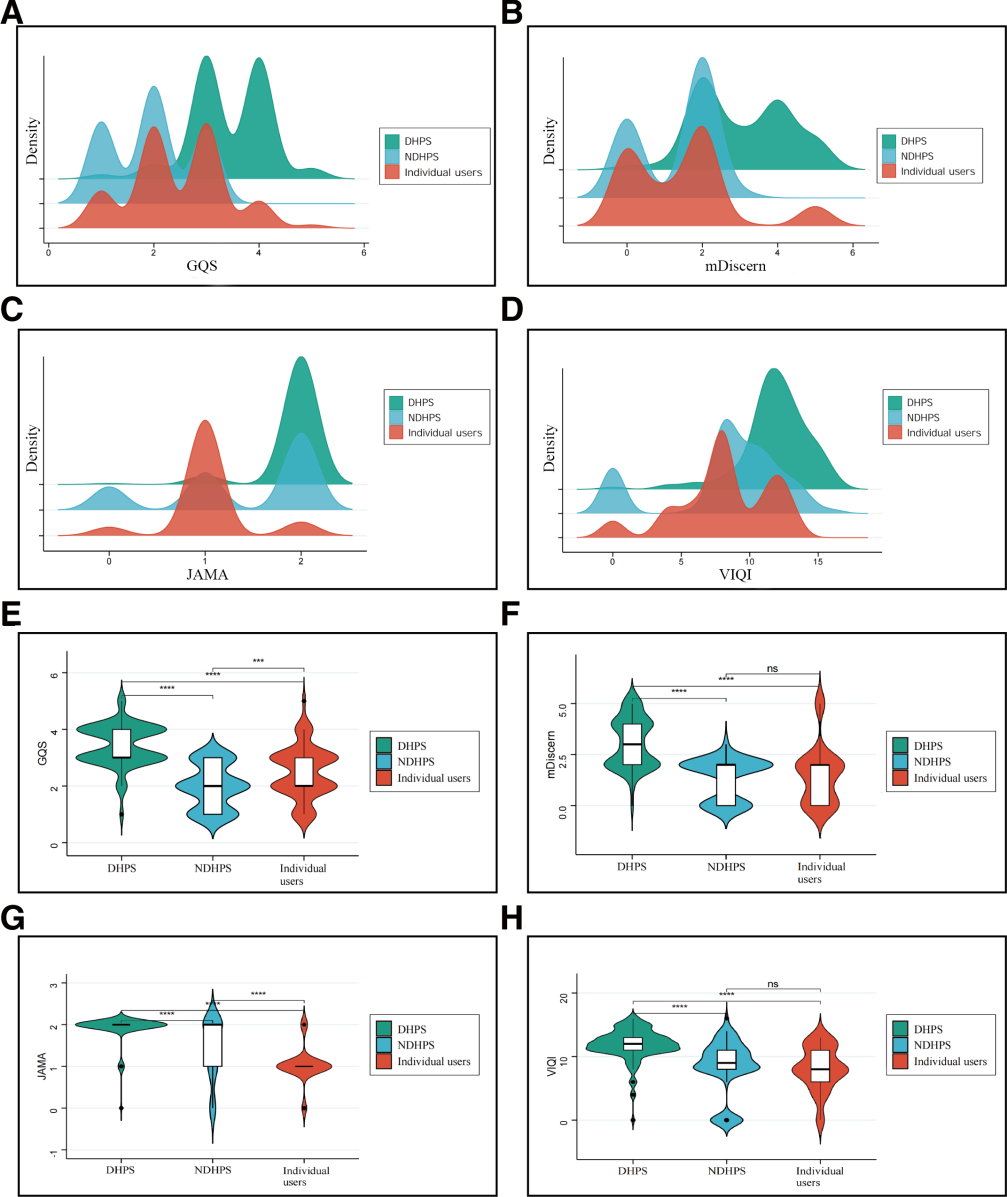
Differences in video quality and reliability scores across uploader types. (A–D) Kernel density distributions of GQS, mDISCERN, JAMA, and VIQI scores across uploader groups, including DHPs, NDHPs, and individual users. (E–H) Violin plots comparing GQS, mDISCERN, JAMA, and VIQI across uploader groups; embedded boxplots indicate the median and interquartile range, and brackets indicate pairwise comparisons (ns, not significant).

Comparisons across uploader types are shown in Table 4 and Figure 5. No significant differences were observed among NDHPs, DHPs, and individual users in video length or engagement indicators, including likes, collections, comments, and shares. By contrast, significant between-group differences were observed for all 4 quality assessment instruments (all *P* < .001).

**Table 4:** Comparison of engagement metrics and quality scores across uploader types.

Variables	NDHPs (n = 77)	DHPs (n = 131)	Individual users (n = 81)	*P*
Video length, s, Median (Q1, Q3)	145.00 (87.00,231.00)	127.00 (86.00,241.00)	165.00 (51.00,414.00)	.772
Likes, Median (Q1, Q3)	274.00 (118.00,4942.00)	696.00 (101.00,5920.50)	239.00 (21.00,3156.00)	.126
Collections, Median (Q1, Q3)	204.00 (75.00,2386.00)	500.00 (56.50,2911.00)	218.00 (33.00, 1113.00)	.261
Comments, Median (Q1, Q3)	20.00 (5.00, 231.00)	41.00 (8.00, 218.00)	24.00 (2.00, 292.00)	.343
Shares, Median (Q1, Q3)	104.00 (18.00, 1502.00)	177.00 (26.00, 934.50)	83.00 (16.00, 1092.00)	.890
GQS score, Median (Q1, Q3)	2.00 (1.00, 3.00)	3.00 (3.00, 4.00)	2.00 (2.00, 3.00)	<.001
mDISCERN score, Median (Q1, Q3)	2.00 (0.00, 2.00)	3.00 (2.00, 4.00)	2.00 (0.00, 2.00)	<.001
JAMA score, Median (Q1, Q3)	2.00 (1.00, 2.00)	2.00 (2.00, 2.00)	1.00 (1.00, 1.00)	<.001
VIQI score, Median (Q1, Q3)	6.00 (3.00, 8.00)	9.00 (7.00, 11.00)	6.00 (3.00, 8.00)	<.001

Continuous variables were compared among uploader types using the Kruskal-Wallis test. When the overall Kruskal–Wallis test was statistically significant, Dunn’s post hoc test with Bonferroni correction was performed for pairwise comparisons. For GQS and JAMA, all pairwise comparisons were statistically significant after correction. For mDISCERN and VIQI, DHP-uploaded videos scored significantly higher than both NDHP-uploaded and individual-user videos, whereas NDHPs and individual users did not differ significantly after correction.

DHPs = doctors of health professions, GQS = Global Quality Score, JAMA = Journal of the American Medical Association, mDISCERN = modified DISCERN, NDHPs = non-doctors of health professions, Q1 = first quartile, Q3 = third quartile, VIQI = Video Information and Quality Index.

Dunn’s post hoc tests with Bonferroni correction showed that DHP-uploaded videos had significantly higher GQS, mDISCERN, JAMA, and VIQI scores than both NDHP-uploaded and individual-user videos. For GQS, all pairwise comparisons were significant after correction, with DHPs showing the highest scores, followed by individual users and NDHPs. For mDISCERN and VIQI, NDHPs and individual users did not differ significantly after correction. For JAMA, all pairwise comparisons were significant, with DHPs scoring highest and individual users lowest.

### 3.5. Correlation analysis of video characteristics and quality

**Figure 6.**
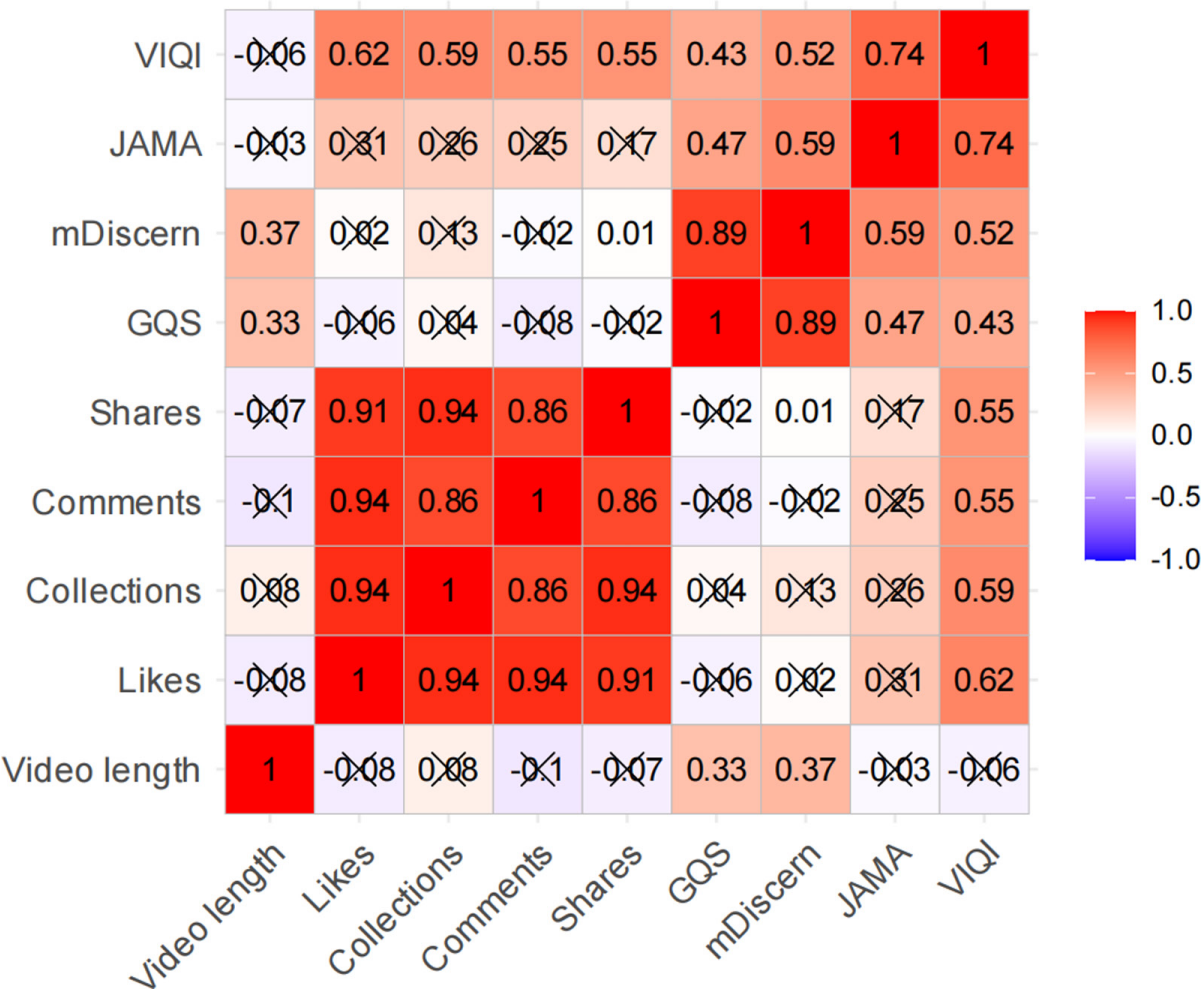
Correlations between engagement metrics, video length, and quality/reliability scores. Heatmap showing correlation coefficients among video length, engagement indicators (likes, collections, comments, and shares), and quality/reliability scores (GQS, mDISCERN, JAMA, and VIQI). Cells marked with × indicate nonsignificant correlations.

The correlation matrix is shown in Figure 6. The 4 quality assessment scores were positively correlated with each other, indicating that videos with higher performance on one quality dimension tended to score higher on the others. Video length showed positive correlations with GQS (*r* = 0.331, *P* < .001), mDISCERN (*r* = 0.375, *P* < .001), and VIQI (*r* = 0.399, *P* < .001), but not with JAMA (*r* = −0.031, *P* = .595). By contrast, correlations between engagement indicators and quality scores were generally weak or nonsignificant, with Spearman coefficients ranging from *r* = −0.130 to *r* = 0.308. Likes, collections, and comments were weakly correlated with JAMA scores (*r* = 0.308, 0.262, and 0.254, respectively; all *P* < .001), whereas most correlations between engagement metrics and GQS, mDISCERN, or VIQI were weak or nonsignificant.

Platform-stratified Spearman analyses showed similarly weak associations between engagement indicators and quality scores. On TikTok, correlation coefficients ranged from *r* = −0.257 to *r* = 0.139, whereas on Bilibili they ranged from *r* = 0.066 to *r* = 0.298 (Supplemental Digital Content 5).

## 4. Discussion

### 4.1. Principal findings

This cross-sectional study evaluated 289 cerebral infarction-related short videos from TikTok and Bilibili and compared their engagement characteristics, content coverage, and information quality across platforms and uploader types. Overall, the findings suggest that cerebral infarction-related short videos have only modest educational quality and substantial gaps in several clinically important domains, particularly epidemiology and diagnosis.^[14,15]^ In addition, engagement indicators were not closely aligned with quality scores, and videos uploaded by doctors of health professions generally showed better quality and reliability than those uploaded by other uploader types.^[16]^ Together, these findings indicate that popularity alone should not be interpreted as a marker of informational value in short-video health communication.

### 4.2. Interpretation of platform differences

Short-video platforms have become increasingly important channels for public exposure to health information, but their role is likely different from that of formal health education materials or clinical consultation. Users may encounter cerebral infarction-related videos not only when actively searching for disease information, but also through algorithmic recommendations, brief topical browsing, or incidental exposure during entertainment-oriented use. Therefore, the audience for such videos may include the general public, family members, caregivers, and individuals with vascular risk factors, but it does not necessarily overlap completely with the population at highest risk of cerebral infarction. This distinction is important when interpreting the function of short-video content: such videos may be particularly valuable for raising awareness, prompting symptom recognition, and encouraging timely care-seeking, but they may be less suitable as a sole source of comprehensive disease education.

The platform comparison showed clear differences in engagement patterns. TikTok videos attracted substantially higher interaction than Bilibili videos, whereas Bilibili videos were longer overall.^[17]^ In practice, shorter and more rapidly consumable content may be more likely to trigger likes, comments, and shares, even when its informational completeness is limited.^[18]^ This may partly explain why engagement indicators were only weakly associated with the quality scores in this study. Accordingly, recommendation systems for health-related videos should be cautious about relying primarily on popularity signals when ranking or promoting content.^[19]^

### 4.3. Content gaps and implications

A notable finding of this study was the highly uneven distribution of content domains. Etiology and clinical manifestations were relatively well represented, whereas epidemiology and diagnosis were rarely covered.^[20]^ This imbalance is important because epidemiology can help viewers understand who is at risk and why prevention matters, whereas diagnosis-related content is directly relevant to recognizing warning signs, understanding the need for urgent evaluation, and navigating appropriate care pathways.^[21]^ Videos that omit these domains may still attract attention, but they may provide limited practical value for early identification and informed action.^[22]^ Several factors may contribute to the underrepresentation of epidemiology and diagnosis. Compared with symptoms, treatment stories, or lifestyle advice, epidemiologic information is often more abstract and may be perceived as less engaging in short-form media.^[23]^ Diagnosis-related content may also be more difficult to present accurately in a brief and accessible format because it often involves clinical reasoning, imaging findings, or distinctions between similar conditions.^[24]^ As a result, creators may preferentially focus on more intuitive or attention-grabbing topics, while platforms may further amplify such content through engagement-driven recommendation mechanisms. This may help explain why some domains with high educational relevance remain underrepresented in the short-video environment.^[2]^

### 4.4. Quality differences across platforms

Regarding quality assessment, differences were observed between TikTok and Bilibili across multiple dimensions (Figs. 3A–H).^[25]^ However, the direction of these differences was not uniform across all instruments. Although TikTok videos showed stronger engagement and higher JAMA scores, Bilibili videos performed better on mDISCERN and VIQI, while GQS differed significantly despite similar median values.^[26]^ These findings suggest that the 2 platforms may differ in specific aspects of information quality, reliability, and presentation rather than showing a simple overall advantage of one platform over the other. In addition, the longer duration of Bilibili videos did not necessarily correspond to consistently better performance across all quality dimensions.^[27]^ This pattern indicates that the dissemination of health videos is influenced not only by platform algorithms, but also by differences in content organization, professionalism, and presentation style.^[28,29]^ From a governance perspective, platform-level measures such as mandatory disclosure of authorship and information sources, the use of structured content templates, review prompts, and quality-aware weighting in health-related search or recommendation scenarios may help improve the completeness of diagnosis-related information and care-seeking guidance.^[30–32]^

### 4.5. Quality differences across uploader types

Videos uploaded by doctors of health professions had consistently higher quality and reliability scores than those uploaded by NDHPs and individual users. This pattern is consistent with the expectation that professional training may improve the accuracy, structure, and transparency of medical communication. However, the higher quality of DHP-uploaded videos did not translate into clearly superior engagement.^[33]^ This suggests that professionally generated health information may face disadvantages in attention competition if it is less optimized for short-video consumption, visual appeal, or platform-native communication styles. From a public health perspective, this mismatch is important: high-quality content may exist on the platform but remain less visible than more engaging, lower-quality alternatives.^[29]^ These findings imply that improving short-video health communication should not rely solely on encouraging professionals to produce content. Platform-level mechanisms may also be needed to increase the discoverability of credible videos, such as clearer source disclosure, verification of professional identity, prioritization of trustworthy health content in search contexts, and interface cues that help users distinguish educational content from entertainment-oriented or weakly sourced videos.^[34,35]^ At the same time, health professionals may need to adapt communication strategies to short-video formats by improving clarity, pacing, visual structure, and retention of key messages without sacrificing informational accuracy.^[36]^

### 4.6. Correlation between engagement and quality

Another important finding was the weak relationship between engagement metrics and information quality.^[26]^ Videos with higher likes, shares, or comments were not necessarily those with better GQS, mDISCERN, JAMA, or VIQI performance.^[27,37]^ This pattern suggests that platform engagement reflects a mixture of factors beyond educational value, including presentation style, emotional resonance, novelty, title framing, timing, and algorithmic amplification.^[34,38]^ For platforms, it underscores the importance of incorporating credibility-related signals into health content recommendation and search ranking systems. For researchers, it highlights the need to avoid treating engagement as a surrogate measure of informational quality in studies of online health communication.^[39,40]^

### 4.7. Strengths and practical implications

This study contributes to the growing literature on health information quality in short-video environments by providing a cross-platform comparison focused specifically on cerebral infarction, a condition in which early recognition and timely response are especially important. By combining platform comparison, uploader-type comparison, content-domain coding, and multiple complementary quality instruments, the study provides a multidimensional view of how cerebral infarction-related information is presented and circulated in Chinese short-video platforms. These findings may inform future efforts to improve digital health communication through better creator guidance, more transparent sourcing practices, and platform-level strategies that do not depend exclusively on popularity-based ranking.

### 4.8. Limitations

This study has several limitations. First, only TikTok and Bilibili were included, and other major platforms were not examined; therefore, the findings may not be generalizable to all short-video ecosystems. Second, the sample was restricted to the first 150 search results from each platform under one predefined keyword strategy, which may not fully capture the broader range of cerebral infarction-related content available online. In particular, related search terms such as “stroke” were not included, which may have led to omission of some relevant videos. Third, as with other content analysis studies, the assessment of video quality and content coverage involved a degree of subjective judgment, although standardized procedures, rater training, and adjudication were used to improve consistency. Fourth, the study was cross-sectional and descriptive; therefore, it cannot determine how exposure to specific video characteristics influences user understanding, behavior, or clinical outcomes. Fifth, no adjusted multivariable analyses were performed. Therefore, potential confounding by platform characteristics, uploader type, video duration, and engagement level could not be fully controlled. In addition, although nonparametric comparisons and correlation coefficients were reported, formal adjusted effect-size estimates were not calculated. Therefore, the between-group differences should be interpreted as exploratory and descriptive rather than as independently adjusted associations.

## 5. Conclusion

Taken together, these findings suggest that cerebral infarction-related short videos on TikTok and Bilibili provide an important but imperfect source of public health information. The main concern is not simply variable quality, but the coexistence of modest overall quality, incomplete coverage of high-value domains, and weak alignment between engagement and informational reliability. Future efforts should therefore focus on improving both the quality and visibility of evidence-informed cerebral infarction content, while adapting communication strategies to the realities of short-video consumption.

## Acknowledgments

The authors thank all the researchers involved in video screening, data extraction, and quality assessment for their contributions to this study. No professional writing assistance was used for the preparation of this manuscript.

## Author contributions

**Conceptualization:** Yifeng Xie, Haiyan Song, Chunyan Chen.

**Data curation:** Yifeng Xie, Yuzhang Liang, Yike Liu, Ya Na, Jiamin Gao, Ao Ying.

**Formal analysis:** Yifeng Xie, Chunyan Chen.

**Investigation:** Yifeng Xie, Yuzhang Liang, Yike Liu, Ya Na, Jiamin Gao, Ao Ying.

**Methodology:** Yifeng Xie, Yuzhang Liang, Haiyan Song, Chunyan Chen.

**Project administration:** Chunyan Chen.

**Supervision:** Haiyan Song, Chunyan Chen.

**Validation:** Yifeng Xie, Haiyan Song, Chunyan Chen.

**Visualization:** Yifeng Xie, Yuzhang Liang.

**Writing – original draft:** Yifeng Xie.

**Writing – review & editing:** Yifeng Xie, Haiyan Song.










