# Supplementary material for: Comparative assessment of the quality and reliability of cerebral infarction–related short-video health information on TikTok and Bilibili: A cross-sectional study
Source: Medicine (Baltimore). 2026 Jun 12;105(24):e49206. doi: 10.1097/MD.0000000000049206 (PMC13268501; doi:10.1097/MD.0000000000049206)
Supplement: Supplementary file 2 [file medi-105-e49206-s002.doc]

**Supplemental Digital Content 2. Modified DISCERN criteria.**

| **Reliability Score** |
| --- |
| 1. Is the video clear, concise, and understandable? |
| 2. Are valid sources cited? |
| 3. Is the content presented balanced and unbiased? |
| 4. Are additional sources of content listed for patient reference? |
| 5. Are areas of uncertainty mentioned? |

Abbreviations: mDISCERN, modified DISCERN.
