# Supplementary material for: Comparative assessment of the quality and reliability of cerebral infarction–related short-video health information on TikTok and Bilibili: A cross-sectional study
Source: Medicine (Baltimore). 2026 Jun 12;105(24):e49206. doi: 10.1097/MD.0000000000049206 (PMC13268501; doi:10.1097/MD.0000000000049206)
Supplement: Supplementary file 3 [file medi-105-e49206-s003.doc]

**Supplemental Digital Content 3. JAMA benchmark criteria for online health information.**

| Score | Score component | |
| --- | --- | --- |
| 1 | Authorship | Author and contributor credentials and their affiliations should be provided. |
| 1 | Attribution | Clearly lists all copyright information and states references and sources for content. |
| 1 | Currency | Initial date of posted content and subsequent updates to content should be provided. |
| 1 | Disclosure | Conflicts of interest, funding, sponsorship, advertising, support, and video ownership  should be fully disclosed. |

Abbreviations: JAMA, Journal of the American Medical Association.
