# Supplementary material for: Comparative assessment of the quality and reliability of cerebral infarction–related short-video health information on TikTok and Bilibili: A cross-sectional study
Source: Medicine (Baltimore). 2026 Jun 12;105(24):e49206. doi: 10.1097/MD.0000000000049206 (PMC13268501; doi:10.1097/MD.0000000000049206)
Supplement: Supplementary file 4 [file medi-105-e49206-s004.doc]

**Supplemental Digital Content 4. Video Information and Quality Index criteria.**

| **Domain** | **Description** |
| --- | --- |
| **Information flow** | Assesses whether the video presents information coherently and logically, in a reasonable order, and is easy for viewers to follow. |
| **Information accuracy** | Assesses whether the information is accurate, evidence-based, and supported by authoritative sources, and whether the content contains errors or is misleading. |
| **Video quality** | Assesses production and presentation quality, including the use of supportive elements (e.g., images, animations, interviews, captions/subtitles, and summaries). Each element contributes 1 point (maximum 5 points). |
| **Precision** | Assesses consistency between the video title and content (i.e., whether the content matches what is promised by the title and whether discrepancies are present). |

Abbreviations: VIQI, Video Information and Quality Index.
