# Supplementary material for: Comparative assessment of the quality and reliability of cerebral infarction–related short-video health information on TikTok and Bilibili: A cross-sectional study
Source: Medicine (Baltimore). 2026 Jun 12;105(24):e49206. doi: 10.1097/MD.0000000000049206 (PMC13268501; doi:10.1097/MD.0000000000049206)
Supplement: Supplementary file 5 [file medi-105-e49206-s005.doc]

**Supplemental Digital Content 5. Spearman correlations between video characteristics, engagement metrics, and quality/reliability scores, stratified by platform.**

| **Group** | **n** | **Metric** | **GQS** | **mDISCERN** | **JAMA** | **VIQI** |
| --- | --- | --- | --- | --- | --- | --- |
| Overall | 289 | Video length | r = 0.331, P < .001 | r = 0.375, P < .001 | r = -0.031, P = .595 | r = 0.399, P < .001 |
| Overall | 289 | Likes | r = -0.059, P = .317 | r = 0.024, P = .679 | r = 0.308, P < .001 | r = -0.100, P = .091 |
| Overall | 289 | Collections | r = 0.044, P = .458 | r = 0.129, P = .029 | r = 0.262, P < .001 | r = 0.035, P = .553 |
| Overall | 289 | Comments | r = -0.076, P = .195 | r = -0.018, P = .760 | r = 0.254, P < .001 | r = -0.130, P = .028 |
| Overall | 289 | Shares | r = -0.025, P = .673 | r = 0.011, P = .850 | r = 0.173, P = .003 | r = -0.057, P = .332 |
| TikTok | 146 | Video length | r = 0.289, P < .001 | r = 0.446, P < .001 | r = 0.361, P < .001 | r = 0.376, P < .001 |
| TikTok | 146 | Likes | r = 0.005, P = .948 | r = 0.104, P = .210 | r = -0.123, P = .140 | r = 0.017, P = .841 |
| TikTok | 146 | Collections | r = 0.018, P = .833 | r = 0.139, P = .095 | r = -0.036, P = .665 | r = 0.051, P = .541 |
| TikTok | 146 | Comments | r = 0.033, P = .691 | r = 0.087, P = .297 | r = -0.195, P = .019 | r = 0.022, P = .793 |
| TikTok | 146 | Shares | r = -0.028, P = .735 | r = -0.004, P = .962 | r = -0.257, P = .002 | r = -0.062, P = .460 |
| Bilibili | 143 | Video length | r = 0.235, P = .005 | r = 0.274, P < .001 | r = 0.170, P = .042 | r = 0.282, P < .001 |
| Bilibili | 143 | Likes | r = 0.201, P = .016 | r = 0.204, P = .014 | r = 0.206, P = .014 | r = 0.185, P = .027 |
| Bilibili | 143 | Collections | r = 0.287, P < .001 | r = 0.298, P < .001 | r = 0.246, P = .003 | r = 0.289, P < .001 |
| Bilibili | 143 | Comments | r = 0.099, P = .238 | r = 0.084, P = .317 | r = 0.128, P = .128 | r = 0.066, P = .435 |
| Bilibili | 143 | Shares | r = 0.159, P = .058 | r = 0.132, P = .115 | r = 0.092, P = .274 | r = 0.139, P = .097 |

**Abbreviations:** GQS, Global Quality Score; mDISCERN, modified DISCERN; JAMA, Journal of the American Medical Association; VIQI, Video Information and Quality Index.

**Note:** Values are Spearman correlation coefficients with corresponding two-sided P values. P values less than 0.001 are displayed as <.001. Overall analysis included 289 videos; platform-stratified analyses included 146 TikTok videos and 143 Bilibili videos.
